# Integration of Clinical and CT-Based Radiomic Features for Pretreatment Prediction of Pathologic Complete Response to Neoadjuvant Systemic Therapy in Breast Cancer

**DOI:** 10.3390/cancers14246261

**Published:** 2022-12-19

**Authors:** Huei-Yi Tsai, Tsung-Yu Tsai, Chia-Hui Wu, Wei-Shiuan Chung, Jo-Ching Wang, Jui-Sheng Hsu, Ming-Feng Hou, Ming-Chung Chou

**Affiliations:** 1 Graduate Institute of Clinical Medicine, College of Medicine, Kaohsiung Medical University, Kaohsiung 807, Taiwan; 2 Department of Medical Imaging, Kaohsiung Medical University Hospital, Kaohsiung Medical University, Kaohsiung 807, Taiwan; 3 Center for Big Data Research, Kaohsiung Medical University, Kaohsiung 807, Taiwan; 4 Department of Medical Imaging, Kaohsiung Municipal Siaogang Hospital, Kaohsiung 812, Taiwan; 5 Department of Biomedical Science and Environmental Biology, College of Life Science, Kaohsiung Medical University, Kaohsiung 807, Taiwan; 6 Department of Medical Imaging and Radiological Science, Kaohsiung Medical University, Kaohsiung 807, Taiwan; 7 Department of Medical Research, Kaohsiung Medical University Hospital, Kaohsiung 807, Taiwan

**Keywords:** breast neoplasm, neoadjuvant therapy, computed tomography, radiomics, machine learning

## Abstract

**Simple Summary:**

This study examined the potential of a machine learning model with integrated clinical and CT-based radiomics features in predicting the pathologic complete response (pCR) to neoadjuvant systemic therapy (NST) in patients with breast cancer. Our results demonstrated that integration of clinical data and radiomics features could significantly improve model performance with accuracy up to 0.87, compared to clinical (0.69) and radiomics (0.78) models. Moreover, the model performance could be further improved by using more high-order textural features with high reproducibility. We concluded that the integration of clinical and CT-based radiomics features was helpful in the pretreatment prediction of pCR to NST in breast cancer.

**Abstract:**

The purpose of the present study was to examine the potential of a machine learning model with integrated clinical and CT-based radiomics features in predicting pathologic complete response (pCR) to neoadjuvant systemic therapy (NST) in breast cancer. Contrast-enhanced CT was performed in 329 patients with breast tumors (*n* = 331) before NST. Pyradiomics was used for feature extraction, and 107 features of seven classes were extracted. Feature selection was performed on the basis of the intraclass correlation coefficient (ICC), and six ICC thresholds (0.7–0.95) were examined to identify the feature set resulting in optimal model performance. Clinical factors, such as age, clinical stage, cancer cell type, and cell surface receptors, were used for prediction. We tried six machine learning algorithms, and clinical, radiomics, and clinical–radiomics models were trained for each algorithm. Radiomics and clinical–radiomics models with gray level co-occurrence matrix (GLCM) features only were also built for comparison. The linear support vector machine (SVM) regression model trained with radiomics features of ICC ≥0.85 in combination with clinical factors performed the best (AUC = 0.87). The performance of the clinical and radiomics linear SVM models showed statistically significant difference after correction for multiple comparisons (AUC = 0.69 vs. 0.78; *p* < 0.001). The AUC of the radiomics model trained with GLCM features was significantly lower than that of the radiomics model trained with all seven classes of radiomics features (AUC = 0.85 vs. 0.87; *p* = 0.011). Integration of clinical and CT-based radiomics features was helpful in the pretreatment prediction of pCR to NST in breast cancer.

## 1. Introduction

Neoadjuvant systemic therapy (NST) is widely accepted as the standard treatment for both locally advanced and early-stage breast cancer [1]. The main goal of NST is to downsize the tumor and ideally achieve pathologic complete response (pCR). pCR is a surrogate endpoint, indicating an event-free and overall survival [2]. A meta-analysis reported that the overall pCR rate was approximately 19%, and there were considerable variations in treatment responses among different tumor subtypes [3]. Breast cancer is known to have a spectrum of biologically distinct subsets leading to diverse tumor behaviors. Recent research has focused on personalized therapy by escalating and de-escalating treatment in non-responders and excellent responders, respectively [4,5]; however, a reliable method to stratify patients for treatment adjustment is still lacking.

Several potential imaging biomarkers for predicting pCR before NST have been assessed. Although a few mammographic or ultrasound features have been associated with pCR [6,7], pCR prediction using conventional images is limited. Antunovic et al. [8] and Li et al. [9] reported that pretreatment positron emission tomography/computed tomography (PET/CT) radiomics features were potential predictors of pCR, and the area under the receiver operating characteristic curve (AUC) ranged from 0.70 to 0.73. Several studies have also investigated the value of breast MRI for the a priori prediction of treatment response to NST, where a combination of radiomics and deep learning methods was used to build prediction models. However, the results varied significantly, with AUC ranging from 0.52 to 0.98 [10,11,12,13,14,15,16]. Because of the variations in pulse sequence selection, image acquisition, reconstruction parameters, and feature extraction, the performance of MR-based prediction models is limited due to a lack of reproducibility and generalizability.

In current practice, breast MRI and PET/CT are not routinely performed in breast cancer patients. However, contrast-enhanced chest CT is included in the preliminary examinations for the detection of possible breast cancer metastasis prior to NST [1]. Although tissue contrast of CT is lower than that of MRI, radiomics, a series of image segmentation, feature extraction, and machine learning analysis, is considered to hold potential for detecting important image information that radiologists are unable to visualize [17,18]. Moghadas-Dastjerdi et al. [19,20] first extracted GLCM-based features of pretreatment contrast-enhanced CT in 72 patients, followed by machine learning to predict tumor response to NST, obtaining AUC values of 0.89 and 0.88 for two Adaboost decision tree models with different feature sets. Their results confirmed the significant potential of CT-based radiomics in predicting breast tumor response to treatment, but the effect of adding more textural features, such as gray-level dependence matrix (GLDM)-, gray-level run length matrix (GLRLM)-, gray-level size zone matrix (GLSZM)-, or neighboring gray tone difference matrix (NGTDM)-based features, into the prediction model remains unclear. Recently, Huang et. al. [21] further extracted size, shape, texture, and wavelet features from pretreatment contrast-enhanced CT in 215 patients to predict treatment response, and they found that the radiomics model could outperform the clinical model (AUC, 0.818 vs. 0.756).

However, due to the lack of standardized definitions of radiomics features and consistent implementation processes, reproducibility and validation of radiomics remain difficult, thereby hindering its clinical use [22]. Furthermore, it remains unknown whether the integration of clinical and radiomic features could outperform clinical and radiomics models in predicting the pCR of breast cancer. We hypothesized that the integration of clinical and additional textural features with high reproducibility would improve the performance of model prediction for breast cancer in response to treatment. Therefore, we examined the potential of CT-based radiomics integrated with clinical features in predicting breast cancer response to NST using a standardized quantitative radiomics tool [23] with five categories of textural features in addition to first-order and shape-based features.

## 2. Materials and Methods

This single-centered, retrospective study was approved by our Institutional Review Board, and the requirement for written informed consent was waived [KMUHIRB-E(I)-20200406].

### 2.1. Study Subjects

**Figure 1:**
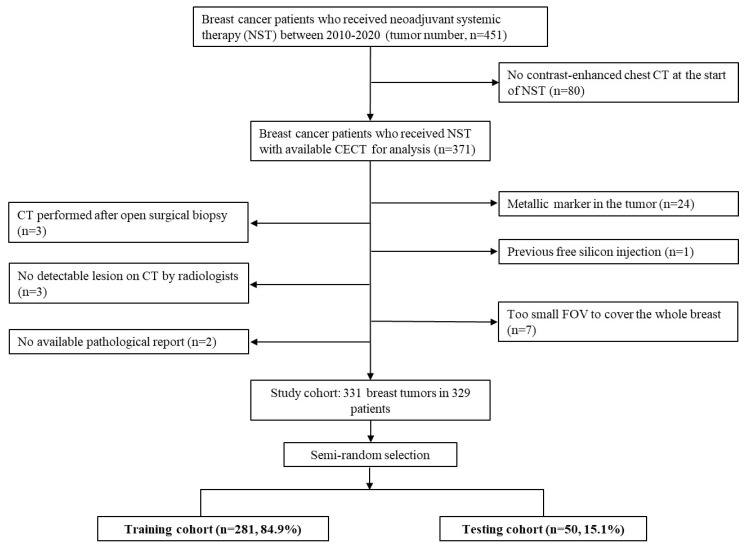
Flowchart of data collection and partition. Data partition was performed using a semi-random selection. The whole dataset was firstly separated into the pCR (+) and pCR (−) groups, followed by random selection of 15% data from the two groups. pCR: pathologic complete response; CECT: contrast-enhanced computed tomography; CT: computed tomography; FOV: field of view.

From January 2010 to December 2020, 451 consecutive breast cancer patients who received NST were enrolled. All patients received treatment regimens according to practice guidelines [1], and the flowchart of subject inclusion is shown in Figure 1. Only patients with contrast-enhanced chest CT performed near the start of NST (within 2 months prior to NST or within 1 day after NST) were included. Patients were excluded if they had (a) metallic markers in the tumor or siliconomas/implants in the breast, (b) CT performed after open surgical biopsy, (c) tumors that could not be delineated by radiologists, (d) incomplete tumor on the image due to a too small field of view (FOV), or (e) no available pathological reports. Ultimately, 331 breast tumors in 329 patients were included in the final study cohort.

### 2.2. Histopathology Evaluation and Clinical Feature Collection

pCR was defined as no residual invasive or in situ cancer cells in the specimen after NST. Six clinical features were collected for model building: age, clinical staging, cancer cell type (ductal, lobular, etc.), estrogen receptor (ER), progesterone receptor (PR), and human epidermal growth factor receptor 2 (HER2). These data were obtained from electronic medical records, including pathology reports. Breast cancer can be classified into four subtypes according to hormone receptor (HR) and HER2 expressions: HR-positive/HER2-negative, HR-positive/HER2-positive, pure HER2-positive, and triple-negative.

### 2.3. Image Acquisition

CT images were acquired from the Picture Archiving and Communication System of our institution and were saved in Digital Image and Communication in Medicine format. Because our institution is an academic medical center with many patients referred from several affiliated hospitals, imaging data were obtained from different scanners (Siemens SOMATOM Definition, SOMATOM Definition AS, SOMATOM Definition Flash, and Sensation 64; GE Medical System Optima CT 660, Discovery CT 750 HD, BrightSpeed S, and LightSpeed 16; Philips Brilliance 64; Toshiba Aquilion, Aquilion ONE, and Aquilion PRIME). The contrast-enhanced CT images were obtained after intravenous iodine contrast injection and reconstructed using a 5 mm slice thickness without gap.

### 2.4. Image Segmentation and Feature Extraction

**Figure 2:**
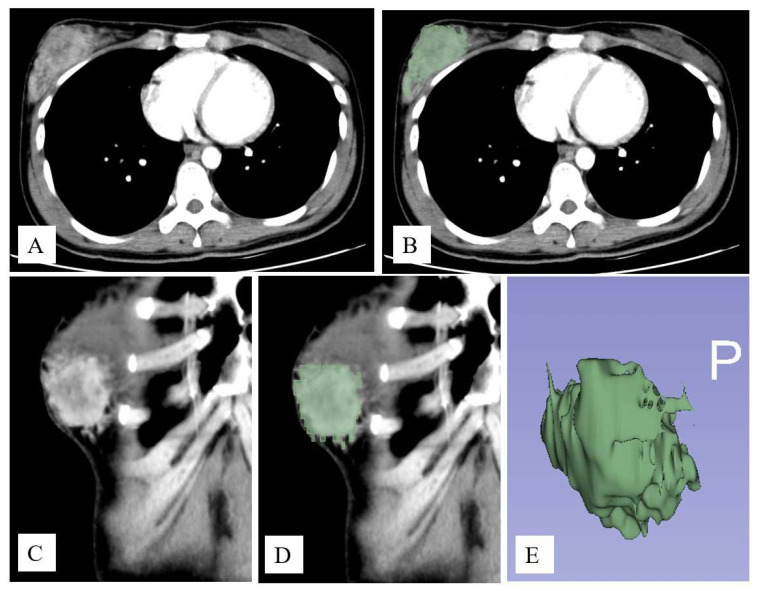
An example of image segmentation. Contrast-enhanced axial (**A**) and coronal (**C**) images show an enhanced tumor in the right breast. Tumor segmentation was performed using the GrowCut semi-automatic segmentation method of 3D-Slicer software (**B**,**D**). The 3D contour of the tumor is shown in (**E**).

Breast tumor segmentation was performed on the basis of axial images using the GrowCut semi-automatic segmentation method of 3D-Slicer software (Slicer 4.11.20210226) [24] (Figure 2). Images were first processed by a third-year resident (T.Y.T.) in the radiology department and further confirmed by two radiologists with consensus agreement (H.Y.T. and C.H.W, with 17 and 4 years of experience, respectively). Radiomic features were automatically extracted using Pyradiomics, an open-source python package, v.3.0.1 [23]. A total of 107 features of seven feature classes were extracted for each tumor, including 14 shape features, 18 first-order features, and 75 textural features (24 GLCM, 14 GLDM, 16 GLRLM, 16 GLSZM, and five NGTDM).

### 2.5. Inter-Rater Reliability and Feature Reproducibility

Before formal image segmentation, radiologists separately performed tumor masking for 20 randomly selected study subjects. Inter-rater reliability was calculated using the Dice coefficient [25]. Dice scores lower than 0.7 were resolved with consensus by a third radiologist (J.S.H., with 25 years of experience). Use of a semi-automatic segmentation tool rendered the concordance of lesion segmentation good, with average Dice scores of 0.86 ± 0.08. The reproducibility of radiomic features was examined by calculating the intraclass correlation coefficient (ICC) of two volumes of interest masked by the two radiologists for the same lesion.

### 2.6. Feature Selection and Model Building

We subdivided the study population into training (*n* = 281, 84.9%) and testing cohorts (*n* = 50, 15.1%). The testing set was randomly selected using a semi-random selection, which firstly distinguished the dataset into pCR(+) and pCR(−) groups and randomly selected 15% data from the two groups, rendering the distribution of the important clinical factors similar to the training dataset [26]. The synthetic minority over sampling technique (SMOTE) was applied to the training dataset to balance pCR data [27,28]. Six machine learning algorithms were examined: linear support vector machine (SVM), Gaussian SVM, polynomial SVM, ensemble learning (Bootstrap aggregation), random forest, and least absolute shrinkage and selection operator (LASSO) regression. For each algorithm, three models, i.e., clinical, radiomics, and clinical–radiomics integration, were trained. All six clinical information sets (age, clinical staging, cancer cell type, ER, PR, and HER2) were used for model building. Radiomic features were selected on the basis of their ICC. We tried six ICC thresholds (0.7, 0.75, 0.8, 0.85, 0.9, and 0.95) to identify the best model performance. To verify our hypothesis, we also built radiomics models using only GLCM features for comparison. Ten-fold cross validation was performed for model hyperparameters using the training set only, and the model performance was examined by the independent testing set. Moreover, an out-of-bag permutation test was carried out to examine the feature importance in the best model. Machine learning was performed using the machine learning toolbox running on the MATLAB platform (MathWorks, Natick, MA, USA).

### 2.7. Statistics

The Pearson χ^2^ test and independent sample *t*-test were used to examine differences between the training and testing sets. The relationship between breast cancer subtype and pCR was assessed using the Pearson χ^2^ test. Sensitivity, specificity, and AUC were the model performance metrics. The DeLong test [29] was used to assess significance of the AUC difference among the three machine learning models (clinical, radiomics, and clinical–radiomics). Analyses were implemented using SPSS (version 22.0, IBM). A *p*-value < 0.05 was considered to indicate a significant difference. Bonferroni correction was used for multiple comparisons.

## 3. Results

### 3.1. Characteristics of the Study Population

The demographic, clinical, and image characteristics of the study population are shown in Table 1. The overall pCR rate was 17.2%. Furthermore, 68.0% (225/331) of tumors were hormone receptor-positive with or without HER2 overexpression. HER2-positive tumors constituted 40.2% (133/331) and triple-negative tumors constituted 15.7%. The average of the interval between CT scanning and the start of neoadjuvant therapy was 6.0 ± 8.6 days. A large proportion (85.1%) of the images were obtained from single institutions, but the CT scanner models were relatively diverse. There was no statistically significant difference in the important variables between training and testing datasets.

Table 2 presents the relationship between cancer subtype and pCR. The HR-positive/HER2-negative group had the lowest pCR rate (6.2%), and the pure HER2-positive group had the highest one (33.3%).

### 3.2. Model Performance

Of the six machine learning algorithms, the random forest and linear SVM were the best models, with AUC values of 0.70 (95% CI: 0.69, 0.70) and 0.69 (95% CI: 0.68, 0.70), respectively, in the testing set. Although sensitivities were low (0.50 and 0.53), their specificities reached 0.81 (95% CI: 0.80, 0.82) and 0.77 (95% CI: 0.74, 0.8), respectively (Table 3).

**Figure 3:**
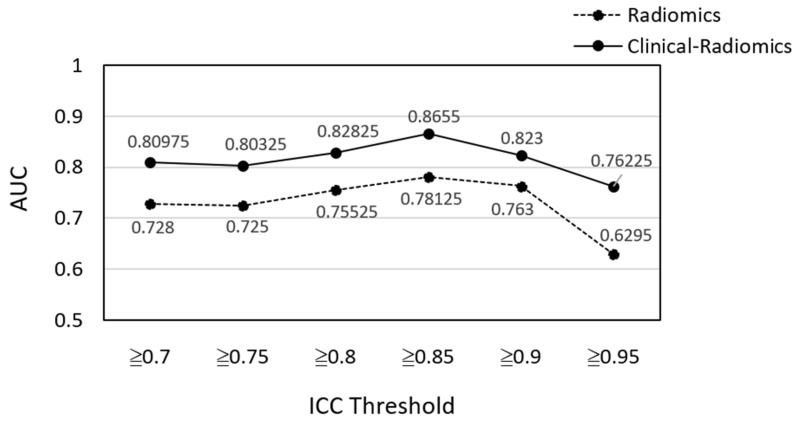
Linear SVM model performance of different radiomic feature sets selected on the basis of different ICC thresholds. ICC = intraclass correlation coefficient; AUC: area under the ROC curve.

**Figure 4:**
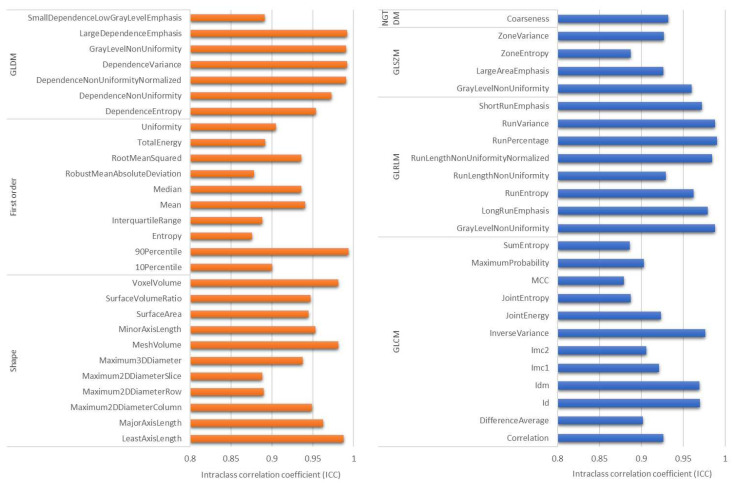
Radiomic features with intraclass correlation coefficient ≥0.85: 11 shape-based, 10 first-order, 12 GLCM, seven GLDM, eight GLRLM, four GLSZM, and one NGTDM features.

Feature selection based on ICC was first performed to train the radiomics model. Six ICC thresholds were tested, and most machine learning models had better performance when features with ICC ≥0.85 were used (Figure 3). There were 53 (49.5%) features with ICC ≥0.85, consisting of 11 shape-based, 10 first-order, 12 GLCM, seven GLDM, eight GLRLM, four GLSZM, and one NGTDM features (Figure 4). Furthermore, linear SVM and LASSO performed better than the other classifiers, while linear SVM was the best model (Table 3). The AUC of the linear SVM model was 0.78 (95% CI: 0.77, 0.79) in the testing set. Its sensitivity and specificity were higher than those of the clinical model (0.59 vs. 0.53 and 0.83 vs. 0.77, respectively).

For training the clinical–radiomics integration model, the six clinical factors were all used, and the six ICC thresholds were tested again with the six machine learning algorithms. Results showed that the linear SVM model using radiomics features with ICC ≥ 0.85 was again the best model in predicting pCR. The AUC increased from 0.69 (clinical model) and 0.78 (radiomics model) to 0.87 (95% CI: 0.86, 0.88). Additionally, the sensitivity increased significantly to 0.90 (Table 3).

The DeLong test was used to assess differences in model performance (Table 4). Bonferroni correction was used due to multiple comparisons, and a *p*-value < 0.017 was considered significant. The results showed significant AUC differences among the clinical, radiomics, and integration models in the testing set.

Radiomics and clinical–radiomics integration models were also trained using GLCM features. The linear SVM model built with features of ICC ≥ 0.85 showed the best performance. The AUC of the radiomics model [radiomics (GLCM)] was 0.77 (95% CI: 0.77, 0.79), slightly lower than that of the radiomics model built with all seven classes of features [radiomics (All)] (AUC = 0.78) without statistical significance (Table 5).

**Figure 5:**
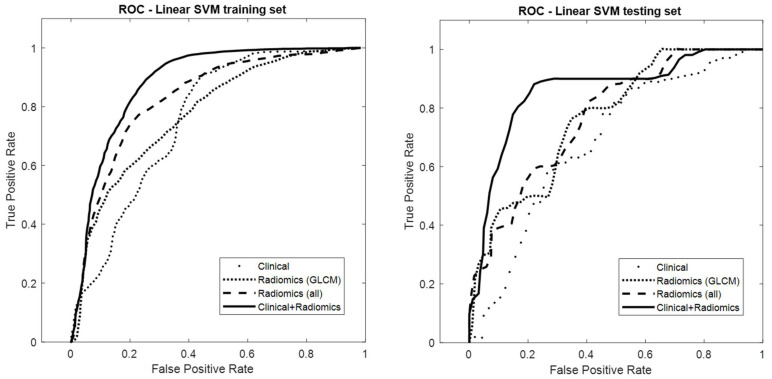
Receiver operating characteristic curves of the clinical, radiomics (GLCM), radiomics (all classes of features), and clinical–radiomics (all classes of features) linear SVM models in the training and testing sets. In the testing set, the AUC values were 0.69, 0.77, 0.78, and 0.87 for clinical, radiomics (GLCM), radiomics (all), and clinical–radiomics (all), respectively.

The performance of the integration model [clinical–radiomics (GLCM)] (AUC = 0.85; 95% CI: 0.84, 0.85) was significantly less accurate than the integration model trained with all classes of features [clinical–radiomics (all)] (AUC = 0.87). Figure 5 presents the ROC curves of the four models: clinical, radiomics (GLCM), radiomics (all), and clinical–radiomics (all).

**Figure 6:**
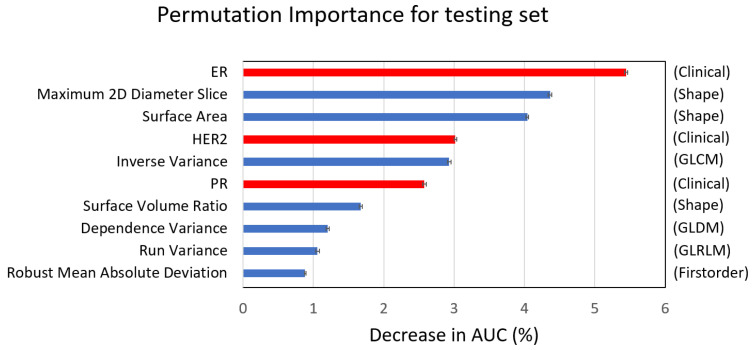
Top 10 important features of the integration linear SVM model, consisting of three clinical (red) and seven radiomics features (blue). ER: estrogen receptor; HER2: human epidermal growth factor receptor 2; PR: progesterone receptor.

Moreover, the permutation importance test showed that the top 10 important features in the linear SVM model consisted of three clinical features (ER, HER2, and PR) and seven radiomics features (three shape, one first-order, one GLCM, one GLDM, and one GLRLM) (Figure 6). It was noted that hormone receptors and tumor shapes were more important features in predicting pCR than high-order textural features (GLCM, GLDM, and GLRLM).

## 4. Discussion

The pCR indicates better long-term outcomes in breast cancer patients receiving NST. Prediction of pCR before NST is valuable for adjusting therapy regimens and selecting patients for clinical trials on de-escalating treatments. In this study, we used routine contrast-enhanced CT and clinical data to build a prediction model with high accuracy (AUC 0.87). Compared to previous studies using MRI (AUC from 0.52 to 0.98) or PET/CT (AUC from 0.70 to 0.73) [8,9,10,11,12,13,14,15,16], pCR could be predicted from the available images without any additional imaging studies, saving significant costs (time and money). Use of a relatively large sample size and the standardized radiomics feature definition and extraction process are additional strengths of this study, rendering this process more reproducible and comparable.

Although contrast-enhanced chest CT is usually performed in breast cancer patients before receiving neoadjuvant therapy, only few studies have used these images to predict treatment response. Moghadas-Dastjerdi et al. [19,20] enrolled 72 patients and confirmed that machine learning models trained with GLCM-based features could predict pCR before treatment. One recent study enrolled 215 patients and performed machine learning modeling of clinical data and CT radiomics to predict pCR. Their results demonstrated that the radiomics model outperformed the clinical model (AUC, 0.818 vs. 0.756) in predicting pCR [22]. However, this study divided datasets into training and testing sets by setting a timepoint, resulting in unmatched clinical features (tumor staging with *p* < 0.05), which may have led to bias. In contrast, the present study enrolled more patients (*n* = 329) and used a semi-random selection, dividing training and testing sets by matching their clinical characteristics, including clinical demographics, pCR rate, hormone receptor, cell type, hospital, and scanners. Moreover, the present study performed SMOTE oversampling to balance pCR(+/−) in the training set, which, together with the matched clinical characteristics between the training and testing sets, may have helped to minimize bias in predicting pCR. Our results further showed that, although the radiomics model with all the classes of features [radiomics (all)] performed slightly better than the model using only GLCM-based features [radiomics (GLCM)] (AUC: 0.78 vs. 0.77, *p* = 0.45), the clinical–radiomics integration model with more radiomics features produced significantly higher performance than that with only GLCM-based features (AUC: 0.87 vs. 0.85, *p* = 0.011). Moreover, the textural features were extracted using Pyradiomics due to its greater completeness of feature categories and standardized process, and several studies showed that machine learning models trained with features extracted from Pyradiomics could predict the neoadjuvant therapy response in metastatic gastric cancer, esophageal cancer, and ovarian cancer [30,31,32]. Our findings indicate that additional texture features have the potential to improve the prediction accuracy for breast cancer response to treatment, especially in the integration model.

Six clinical factors were used to predict pCR [33]. Among them, HR and HER2 were used to classify breast cancer subtype, which was also confirmed to be related to pCR in our study (Table 2). We trained machine learning models using only these clinical features and found a moderate performance (AUC 0.69). Many other clinical and histopathological factors have been related to pCR [33], and the predictive role of biomarkers, such as the 21-gene assay [34], PIK3CA mutation [35], Ki-67 index [36], and tumor-infiltrating lymphocytes [37], is being evaluated. However, because of tumor heterogeneity, biopsy sampling errors occur in biomarkers based on specimens from core needle biopsy. Imaging studies with pixel-based analysis of the whole tumor could compensate for this sampling error. Consequently, the clinical–radiomics integration model performed significantly better than the clinical model in the present study (AUC: 0.87 vs. 0.69). Moreover, the permutation importance test showed that the top 10 features consisted of three clinical features (ER, PR, and HER2) and seven radiomics features (three shape, one first-order, and three texture) in the integration linear SVM model. The findings indicate that hormone receptors and tumor size, shape, and texture were important prognostic features in predicting pCR. However, the relationship between radiomics features and tumor microenvironment, such as tumor-infiltrative lymphocyte or molecular characteristics, still needs further investigation.

Although a standardized feature extraction process was used, the reproducibility of radiomics features is still influenced by many factors, including image acquisition, reconstruction, and segmentation. We used a semi-automatic method to perform image segmentation, which might have increased the reliability of radiomics features [38]; however, the impact of image acquisition and reconstruction on feature reliability was greater than that of inter-observer segmentation [39]. Consequently, ICC was used for feature selection. In general, ICC values of 0.5–0.75 indicate moderate reliability, 0.75–0.9 indicate good reliability, and >0.90 indicate excellent reliability [40]. To date, there is no consensus in radiomics regarding which ICC threshold is suitable for feature selection. Hence, we experimented with six thresholds (0.7–0.95) to identify the feature set resulting in the best model performance. Eventually, 0.85 was determined by data-driven analysis. For ICC ≥0.85, shape and first-order features were more robust than textural features, which is consistent with prior studies [39].

Our study had some limitations. Firstly, this was a retrospective study, and a large portion of patients were enrolled from one institution. The radiomics models remain to be externally validated with images from multiple centers. Secondly, the insufficient sample size did not allow us to perform a deep learning approach to predict pCR, which may have possibly shown better performance than the machine learning approach; however, this was beyond the scope of the present study. Thirdly, the manual tumor segmentation needed for radiomics analysis was relatively time-consuming and labor-intensive, which was potentially slightly reduced by the semi-automatic approach used in this study. Fourthly, the imbalanced pCR(+/−) dataset may affect the performance of models in other datasets with different pCR rates. Further investigation is needed to reduce bias by using datasets with balanced pCR. Lastly, breast cancer is a group of heterogenous diseases with different subtypes and treatment regimens. Training prediction models using a larger study cohort or based on each cancer subtype could be beneficial to clinical practice.

## 5. Conclusions

The present study confirmed that a combination of machine learning models trained with radiomics features derived from pre-NST contrast-enhanced CT and clinical information could accurately predict pCR in breast cancer patients. The model performance could be improved by adding more categories of textural features with a standard feature extraction process. Therefore, we concluded that the integration of clinical and CT-based radiomics features was helpful in the pretreatment prediction of pathologic complete response to NST in breast cancer.

## Figures and Tables

**Table 1:** Numbers of data and comparisons of variables in training and testing datasets (N, %).

	Training	Testing	Total	*p*-Value
**Clinical Variables**	**N = 280, 84.6%**	**N = 51, 15.4%**	**N = 331, 100%**	
pCR ^‡^				0.804
Negative	232 (82.6)	42 (84.0)	274 (82.8)	
Positive	49 (17.4)	8 (16.0)	57 (17.2)	
Age	51.5 ± 9.8	52.5 ± 8.9	51.6 ± 9.7	0.5
Clinical stage				0.741
1	15 (5.3)	1 (2.0)	16 (4.8)	
2	107 (38.1)	21 (42.0)	128 (38.7)	
3	79 (28.1)	13 (26.0)	92 (27.8)	
4	80 (28.5)	15 (30.0)	95 (28.7)	
Breast cancer subtype ^§^				0.951
HR(+), HER2(−)	123 (43.8)	23 (46.0)	146 (44.1)	
HR(+), HER2(+)	68 (24.2)	11 (22.0)	79 (23.9)	
HR(−), HER2(+)	45 (16.0)	9 (18.0)	54 (16.3)	
Triple-negative	45 (16.0)	7 (14.0)	52 (15.7)	
Histology				0.067
Invasive carcinoma of no special type	263 (93.6)	42 (84.0)	305 (92.1)	
Invasive lobular carcinoma	7 (2.5)	3 (6.0)	10 (3.0)	
Others	11 (3.9)	5 (10.0)	16 (4.8)	
Imaging variables	N = 279, 84.8%	N = 50, 15.2%	N = 329, 100%	
Manufacturer				0.971
GE Healthcare	114 (40.9)	20 (40.0)	134 (40.7)	
PHILIPS	48 (17.2)	10 (20.0)	58 (17.6)	
SIEMENS	87 (31.2)	15 (30.0)	102 (31.0)	
TOSHIBA	30 (10.8)	5 (10.0)	35 (10.6)	
Institution				0.533
KMUH ^†^	236 (84.6)	44 (88.0)	280 (85.1)	
Other hospitals	43 (15.4)	6 (12.0)	49 (14.9)	

^†^ KMUH: Kaohsiung Medical University Hospital; ^‡^ pCR: pathological complete response; ^§^ HR: hormone receptor; HER2: human epidermal growth factor receptor 2.

**Table 2:** Relationship between cancer subtype and pathologic complete response (N, %).

Cancer Subtype	Non-pCR (*n* = 274, 82.8)	pCR (N = 57, 17.2)	Total (N = 331, 100)	*p*-Value
HR(+), HER2(−)	137 (93.8)	9 (6.2)	146 (100)	<0.001
HR(+), HER2(+)	60 (75.9)	19 (24.1)	79 (100)	
HR(−), HER2(+)	36 (66.7)	18 (33.3)	54 (100)	
Triple-negative	41 (78.8)	11 (21.2)	52 (100)	

pCR: pathologic complete response; HR: hormone receptor; HER2: human epidermal growth factor receptor 2.

**Table 3:** Performance of clinical, radiomics, and clinical–radiomics models.

Model	Features	Training Dataset			Testing Dataset		
		AUC	Sensitivity	Specificity	AUC	Sensitivity	Specificity
LASSO	Clinical	0.79 (0.78, 0.79)	0.82 (0.80, 0.84)	0.65 (0.63, 0.66)	0.69 (0.68, 0.69)	0.53 (0.44, 0.62)	0.76 (0.73, 0.80)
	Radiomics	0.82 (0.82, 0.83)	0.78 (0.75, 0.80)	0.76 (0.74, 0.78)	0.76 (0.75, 0.77)	0.57 (0.54, 0.60)	0.83 (0.80, 0.86)
	Clinical-Radiomics	0.90 (0.90, 0.90)	0.93 (0.92, 0.95)	0.74 (0.72, 0.76)	0.86 (0.86, 0.87)	0.90 (0.90, 0.90)	0.84 (0.83, 0.85)
Linear SVM	Clinical	0.76 (0.76, 0.77)	0.89 (0.86, 0.91)	0.60 (0.56, 0.63)	0.69 (0.69, 0.70)	0.53 (0.46, 0.60)	0.77 (0.74, 0.80)
	Radiomics	0.83 (0.83, 0.83)	0.76 (0.72, 0.79)	0.79 (0.77, 0.82)	0.78 (0.77, 0.79)	0.59 (0.55, 0.63)	0.84 (0.80, 0.87)
	Clinical-Radiomics	0.88 (0.88, 0.89)	0.90 (0.88, 0.92)	0.75 (0.73, 0.76)	0.87 (0.86, 0.88)	0.90 (0.90, 0.90)	0.82 (0.80, 0.84)

LASSO: least absolute shrinkage and selection operator; SVM: support vector machine; AUC: area under the ROC curve. The numbers in the parentheses are 95% confidence intervals.

**Table 4:** The differences in model performance in testing set examined using DeLong test, represented by *p*-value.

	All Radiomic Features (ICC ≥ 0.85)			GLCM-Based Features (ICC ≥ 0.85)		
Model	Clinical	Radiomics	Clinical–Radiomics	Clinical	Radiomics	Clinical–Radiomics
Clinical	1	<0.001	<0.001	1	<0.001	<0.001
Radiomics		1	<0.001		1	<0.001
Clinical–radiomics			1			1

GLCM: gray-level co-occurrence matrix. A *p*-value < 0.017 was considered significant after Bonferroni correction.

**Table 5:** The effect of adding more textural features to train models for pCR prediction in breast cancer.

Features ^‡^	Model	AUC	Sensitivity	Specificity	*p*-Value ^§^
Radiomics (GLCM)	Linear SVM	0.77 (0.77, 0.79)	0.54 (0.51, 0.57)	0.84 (0.80, 0.88)	0.45
Radiomics (All)	Linear SVM	0.78 (0.77, 0.79)	0.59 (0.55, 0.63)	0.83 (0.80, 0.87)	
Clinical–radiomics (GLCM)	Linear SVM	0.85 (0.84, 0.85)	0.85 (0.79, 0.91)	0.78 (0.74, 0.82)	0.011
Clinical–radiomics (All)	Linear SVM	0.87 (0.86, 0.88)	0.90 (0.90, 0.90)	0.82 (0.80, 0.84)	

GLCM: gray-level co-occurrence matrix. ^‡^ All features or GLCM features with ICC ≥ 0.85. ^§^ Using DeLong test to examine AUC difference between two models.

## Data Availability

Not applicable.
